# *Ambrosia artemisiifolia* L. temperature-responsive traits influencing the prevalence and severity of pollinosis: a study in controlled conditions

**DOI:** 10.1186/s12870-019-1762-6

**Published:** 2019-04-25

**Authors:** Rodolfo Gentili, Riccardo Asero, Sarah Caronni, Maria Guarino, Chiara Montagnani, Gianni Mistrello, Sandra Citterio

**Affiliations:** 1 Department of Earth and Environmental Sciences, University of Milano-Bicocca, Piazza della Scienza 1, 20126 Milano, Italy; 2 Ambulatorio di Allergologia, Clinica San Carlo, Paderno Dugnano, MI Italy; 3 Lofarma SpA, Milan, Italy

**Keywords:** *Ambrosia artemisiifolia*, Amb a 1, Common ragweed, Development, Flavonoids, Pollen allergenicity, Temperature

## Abstract

**Background:**

*Ambrosia artemisiifolia* L. is one of the most important sources of allergenic pollen in many regions of the world. Its health impact increased over the last decades and is expected to further increase in consequence of climate change. However little information is available on the specific role played by temperature on allergy rising. The aim of this work was to evaluate the effect of temperature on *A. artemisiifolia* growth, flowering and pollen allergenicity, the major plant functional traits influencing the prevalence and severity of pollinosis.

**Results:**

Plants were grown in controlled conditions at three thermal regimes: “Low” (LT: 18–14 °C light-dark), “Intermediate” (IT: 24–20 °C light-dark) and “High” (HT: 30–26 °C light-dark). During plant development, plant vegetative and reproductive morpho-functional traits were measured and, at the end of plant life-cycle, mature pollen was collected and analyzed for its allergenic properties by slot blot, 1D- and 2D-western blot (by using a pool of sera from ragweed-allergic patients) and liquid chromatography-tandem mass spectrometry. *A. artemisiifolia* showed a great development plasticity leading to a broad temperature tolerance. Shoot architecture, growth rate, number of male inflorescence and pollen allergenicity were temperature-responsive traits. Pollen allergenicity increased in parallel with temperature and differences were related to allergen synthesis and Amb a 1-IgE-binding. Flavonoids whose concentration in pollen decreased with the increase of temperature, were recognized as the cause of the negligible Amb a 1-IgE binding in LT pollen.

**Conclusions:**

Results show that temperature governs plant development and pollen allergenicity influencing the temporal and spatial magnitude of subject exposure to allergens.

**Electronic supplementary material:**

The online version of this article (10.1186/s12870-019-1762-6) contains supplementary material, which is available to authorized users.

## Background

*Ambrosia artemisiifolia* L. (common ragweed), a North American native species, alien and invasive in Europe, is one of the most important seasonal allergenic plant in many regions of the world [1].

In the United States, its highly allergenic pollen affects more than 36 million people each year and the prevalence of sensitization is growing [2]. Also in Europe the prevalence of ragweed sensitization is relevant and rising. The mean sensitization prevalence is about 14%, with remarkable differences between the countries: it ranges from around 60% in Hungary to 19.5% in Southern Bavaria and it is virtually absent in certain biogeographical regions such as Mediterranean (e.g. Spain), Atlantic (e.g. UK) and Boreal (e.g. Sweden) [3]. In any case, in all other European countries except Finland, the prevalence for ragweed sensitization is above 2.5% that was suggested as a cut-off for high prevalence [4, 5].

A synergy of anthropogenic and bioecological factors was suggested to be responsible for the observed rising prevalence of ragweed sensitization. The globalization of commerce and land use changes have dramatically favored the spread of the species in both America and Europe. At the same time, climate changes have prolonged the ragweed flowering season, and increased the growth of the plant and the pollen production, extending, on the whole, the geographical area and the period/intensity of exposure to its allergens. Indeed, greenhouse experimental simulations of climate change by increasing temperature and/or CO_2_ were demonstrated to determine an earlier flowering, larger floral numbers and a greater pollen production in common ragweed [6, 7]. Similar effects were observed by Ziska et al. [8, 9] and by Rodríguez-Rajo et al. [10] for ragweed plants grown at urban locations where the concentration of CO_2_ and temperature were higher than in rural areas.

Moreover, a few studies suggested that environmental changes can also contribute to the increase of sensitization prevalence by increasing the allergenic potential of ragweed pollen through the modulation of allergen synthesis and structure. Specifically, Ghiani et al. [11] suggested that changes in climatic environmental factors (light, humidity and temperature) during plant development affect the pollen content of the major common ragweed allergen, Amb a 1. In keeping, El Kelish et al. [12] demonstrated that both an elevated level of CO_2_ and drought stress affect *A. artemisiifolia* pollen allergenicity because expressed sequence tags encoding allergenic proteins increased under those conditions. Zhao et al. [13] showed the direct influence of elevated NO_2_ on the increased allergenicity of ragweed pollen and Ghiani et al. [14] demonstrated that traffic-related pollution enhances ragweed pollen allergenicity, showing that pollen collected along high-traffic roads shows a higher whole allergenicity than pollen from low-traffic roads and vegetated areas.

Due to the ongoing global climate change, the current situation is expected to worsen in the next few decades. Species Distribution Models (SDMs) for *A. artemisiifolia* predict that its potential distribution will increase globally [15, 16]. For Europe, Rasmussen et al. [17] suggest that, by the year 2100, the distribution range of ragweed will increase toward Northern and Eastern Europe under all climate scenarios and consequently the high allergy-risk areas will expand in the continent.

Despite this warning, to our knowledge there are no data or very few studies deepen the specific effect of temperature on common ragweed growth and pollen allergenicity. In this work we analyzed the variation in morpho-functional traits, total pollen allergenicity and allergen profile/IgE (Immunoglobulin E) reactivity of ragweed plants germinated and grew in controlled conditions at the following three thermal regimes: “Low” (LT: 18–14 °C light-dark), “Intermediate” (IT: 24–20 °C light-dark) and “High” (HT: 30–26 °C light-dark).

## Results

### Effect of temperature on seed germination and plant development

A trait-based approach for defining the species responses to environmental changes was applied to determine the effect of temperature on plant development. Preliminary germination tests were carried out on agar plates and showed a significantly lower germination (61%) at LT than at IT (81%) and HT (81%). The result was confirmed by evaluating the percentage of germinated seeds in soil before the setup of pot trays with ragweed seedlings (data not shown).

The analysis of functional vegetative and reproductive traits on developing plants showed differences among the three temperature groups. Table 1 shows the mean values of traits, measured at the end of plant development. Although all the plants completed their life cycle, producing a comparable biomass, they showed different shoot architecture at the three thermal regimes. At LT plants were significantly shorter (14.4 ± 4.6 cm) and more laterally expanded (14.5 ± 2.1 cm) than plant grown at IT (37.0 ± 10.5 cm and 11.0 ± 3.2 cm, respectively) and HT (44.7 ± 13.3 cm and 8.6 ± 3.4 cm, respectively). In addition, plants grown at LT showed the highest number of male inflorescences but a significant late flowering (4–5 weeks later than plants grown at IT and HT).

**Table 1:** Measurements (mean ± standard deviation) of vegetative and reproductive traits at the end of *A. artemisiifolia* plant development. LT: Low Temperature, IT: Intermediate Temperature, HT: High Temperature. Different letters means statistical significant differences (*p* < 0.01) between LT, IT and HT for each plant trait

Plant traits	LT		IT		HT	
Germination (%)	61	b	81	a	81	a
Plant height (cm)	14.4 ± 4.6	b	37.0 ± 10.5	a	44.7 ± 13.4	c
Lateral expansion (cm)	14.5 ± 2.1	b	11.0 ± 3.2	ab	8.6 ± 3.4	c
Dry shoot weight (g)	0.6 ± 0.2	b	0.9 ± 0.4	ab	0.5 ± 0.3	b
Number of female flowers plant^− 1^	16.2 ± 15.7	b	22.2 ± 18.8	b	13.8 ± 11.1	b
Number of male racemes plant^− 1^	7.3 ± 3.3	b	4.5 ± 2.3	a	2.3 ± 1.5	c
Start of male flowering (weeks)	13 ± 2.1	b	8 ± 1.1	a	9 ± 1.1	a
Start of female flowering (weeks)	18 ± 1.7	b	13 ± 1.6	ab	13 ± 2.7	ab

### Effect of temperature on pollen allergenicity

The allergenic potential of pollen from plants grown at different temperature was assessed by protein slot blot technique to preserve allergen conformation, on which IgE binding may depend.

**Fig. 1:**
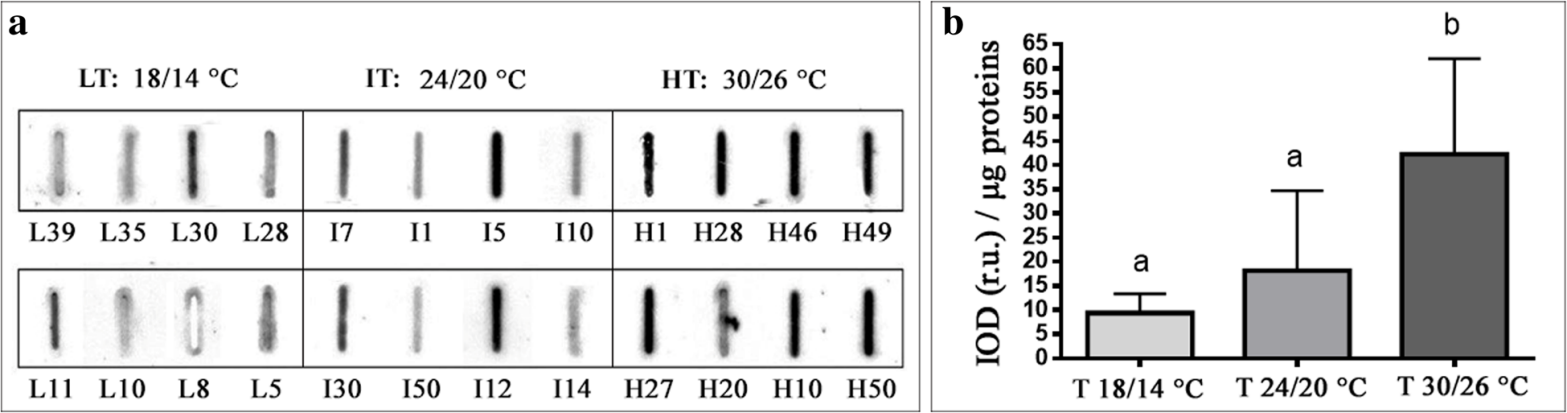
Total allergenicity of pollen collected from *A. artemisiifolia* plants grown at different temperatures. **a** Representative slot blot membrane probed with a pool of selected patient sera. L: samples from plants grown at LT (18–14 °C light-dark); I: samples from plants grown at IT (24–20 °C light-dark); H: samples from plants grown at HT (30–26 °C light-dark). **b**: Quantification of immunochemical (IgE-binding) signals through image analysis: the integrated optical density (IOD) of immunoreactive spots with respect to the IOD of the standard was measured. The results reported are the mean of three independent experiments. Different letters indicate significant differences among the samples (Kruscal-Wallis test, *p* < 0.05)

Identical amounts of proteins from pollen extracts were bound on a nitrocellulose membrane and subjected to immunoreaction with a sera mix from selected ragweed allergic patients. Figure 1a shows a representative membrane after immunodetection. Image analysis was applied to quantify immunochemical signals: the integrated optical density (IOD) of immunoreactive spots with respect to the IOD of standard was measured and results expressed as IOD (sample IOD/standard IOD) related to μg of proteins or g of pollen.

Pollen from single plants was examined and the mean results of three independent experiments were calculated for each temperature and statistically analyzed (Fig. 1b). On average, the highest (42.3 ± 19.2) and lowest (9.4 ± 4.0) IOD/μg proteins values were found for plants grown at HT and LT, respectively. Plants grown at IT showed intermediate values with a mean of 18.1 ± 16.5 IOD/μg proteins. Kruskal-Wallis test showed a statistically significant difference (*p* < 0.05) between HT and the other two groups.

**Fig. 2:**
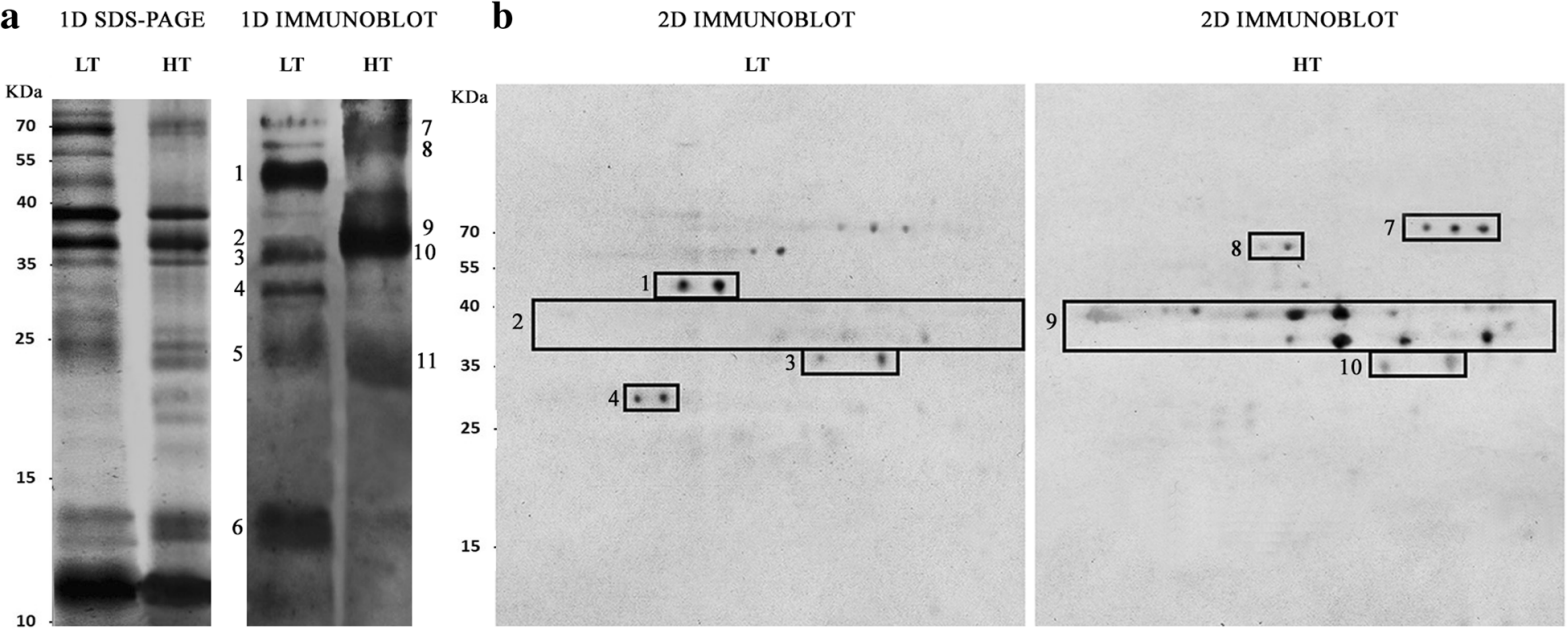
Protein profile and allergen pattern of pollen from plants grown at LT and HT. **a** Representative 1D SDS-PAGEs stained with silver blue to show the pollen protein profiles and the related 1D immunoblot membranes probed with the same sera mix used for slot blot (allergen pattern). **b** Representative 2D immunoblot membranes probed with the same sera mix used for slot blot and 1D immunoblot. IgE bound proteins were identified by LC-MS-MS: (1) Amb a 12 and UDP-glucose pyrophosphorylase-like, (2) Amb a 1.03, (3) the cysteine protease Amb a 11, (4) Desiccation related protein PCC13–62-like, (5) triosephosphate isomerase like protein and Amb a 1.05, (6) Amb a 1 beta chain and Amb a 3, (7) berberine bridge enzyme-like 21, (8) glyoxal oxidase enzyme N-terminus like, (9) Amb a 1 isoforms, (10) Amb a 11, (11) triosephosphate isomerase like protein and Amb a 1.05

To investigate the cause of the difference in slot blot allergenicity, allergen profiles of plants grown at LT and HT were obtained by 1D and 2D-immunoblotting, probed with the same sera mix used for slot blotting. Figure 2 shows representative membranes, where the single allergens, recognized by IgE in the extracts and identified by LC-MS/MS (liquid chromatography-tandem mass spectrometry), can be observed. Allergenic pattern was different between LT and HT samples. In pollen extracts from plants grown at HT, the main IgE bound proteins were all the Amb a 1 isoforms, the cysteine protease Amb a 11 and two proteins (a berberine bridge enzyme-like protein and an oxidase like protein) not yet included in the official IUIS (International Union of Immunological Societies) allergen database, but already identified as IgE reacting proteins by Bordas-Le Floch et al. [18]. An additional protein recognized by the sera mix only in 1D immunoblotting was a triosephosphate isomerase-like protein. In pollen extracts from plants grown at LT, Amb a 11, Amb a 12, a UDP-glucose pyrophosphorylase-like and a desiccation related protein PCC13–62-like were the main IgE bound proteins identified in both 1D and 2D (one-dimensional and two-dimensional) immunoblotting experiments. Notably, only some of the differences in allergen pattern were ascribed to the difference in the presence/amount of proteins in the extracts. This is the case of Amb a 12 (Enolase)/ UDP-glucose pyrophosphorylase-like protein that were detected only in LT extracts and recognized by the sera mix only in those samples. On the contrary, although the Amb a 1 isoforms were similarly present in both LT and HT pollen samples, as indicated by 1D-SDS-PAGE (Sodium Dodecyl Sulphate-PolyAcrylamide Gel Electrophoresis) (Fig. 2), all the isoforms were recognized only in HT samples, whereas in LT samples Amb a 1.03 was the sole IgE bound isoform showing also a faint signal (Fig. 2).

### Flavonoids and allergenicity

**Fig. 3:**
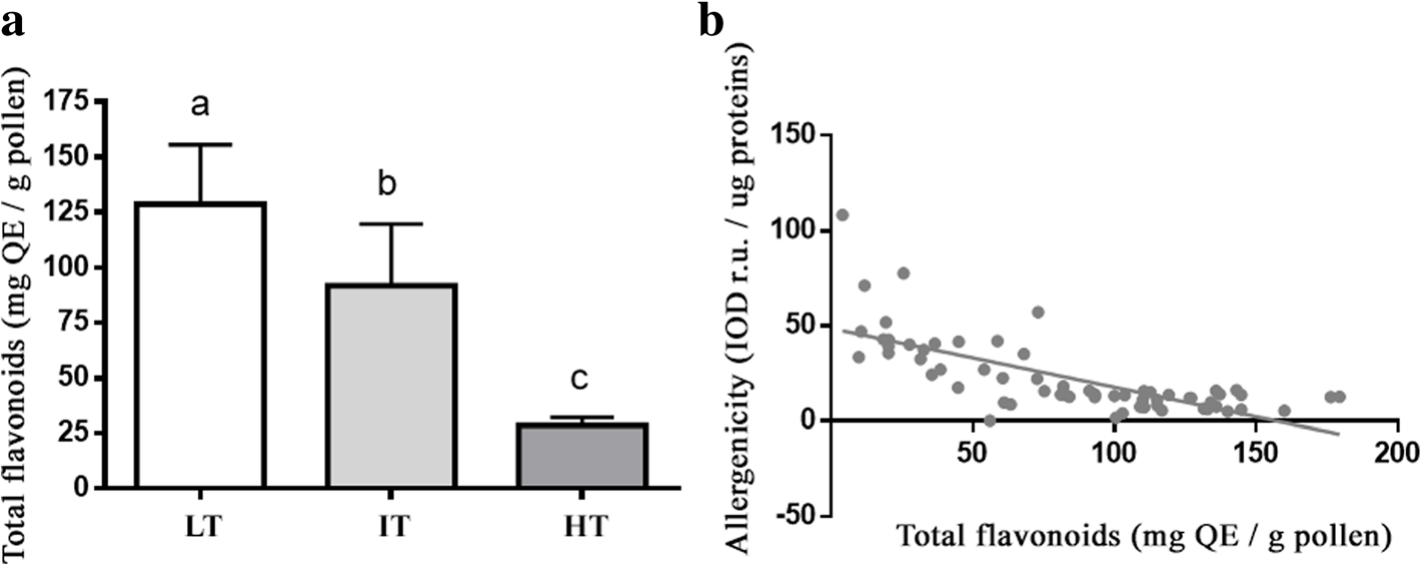
**a** Mean total content of flavonoids in pollen extracts calculated for the three growth temperatures. Different letters indicate significant statistical differences among groups, LT, IT and HT (ANOVA and Tukey test *p* < 0.001); **b** Linear regression analysis between total flavonoids content and total allergenicity (*p* < 0.0001; R^2^ = 0.5198)

Flavonoids were quantified in pollen extracts as their physical interaction with allergenic proteins may limit their IgE binding [19]. The flavonoid content of pollen extracts was determined for each plant (Additional file 1: Figure S1) and the mean value for each temperature was calculated and reported in Fig. 3a. On average, the lowest and highest content of flavonoids was found in HT and LT pollen, respectively (ANOVA, *p* < 0.001). By applying a regression analysis, a quite strong inverse relationship between flavonoids content and total allergenicity (*p* < 0.0001; R^2^ = 0.5198) was observed (Fig. 3b). Specifically, the increase of flavonoid content was related to the decrease of total allergenicity (Y = -0.3090X ± 0.03772).

**Fig. 4:**
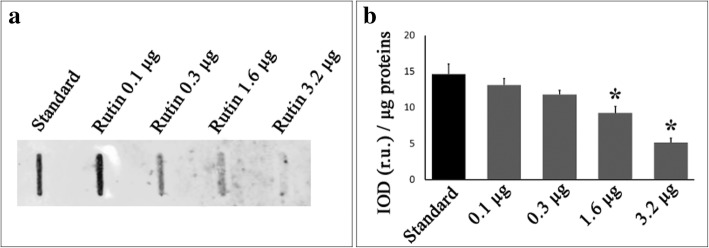
Effect of rutin on *A. artemisiifolia* allergen-IgE binding. **a** Representative slot blot membrane showing the effect of increasing concentrations of rutin on allergen-IgE binding (allergenicity). Increasing amount of rutin (ranging from 0 to 3.2 μg) were added to 2 μl of commercial pollen extract containing 3 μg of proteins, bound to nitrocellulose membrane and treated in order to assess the reaction with the pooled sera as reported in material and methods. **b** Quantification of immunochemical signals through image analysis: the integrated optical density (IOD) of immunoreactive spots was measured and compared with that of the standard (rutin concentration = 0). The results reported are the mean of five independent experiments. * statistically different (ANOVA, *p* < 0.01)

In order to confirm and explain the relationship between flavonoids and IgE binding to allergens, increasing amounts of rutin were added to a commercial pollen extract containing a low amount of flavonoids and showing high allergenicity. The effect of rutin on allergenicity was evaluated with slot blot technique. Results showed that the addition of rutin clearly affected IgE binding (Fig. 4) indicating a direct involvement of flavonoids in modulating pollen allergenicity.

## Discussion

The prevalence and severity of allergic diseases are dependent on both the intrinsic allergenic potency of pollen and the exposure of atopic subjects to allergens.

Temperature is widely recognized as one of the major determinants of plant distribution and pollen production determining the temporal and spatial magnitude of the exposure. In our experiment *A. artemisiifolia* showed a great development plasticity leading to a broad temperature tolerance. All germinated seeds developed in plants showing thermomorphogenic changes of shoot architecture useful for facing temperature changes. Indeed, as observed in the model species *Arabidopsis thaliana*, ragweed growth at HT resulted in striking elongation of stems and increased leaf elevation from the soil surface, that are architectural adaptations representing a strategy to minimize heat damages by enhancing evaporative leaf cooling in well-watered environments [20, 21]. This is in agreement with the study of Bazzaz [22] on ragweed photosynthesis, who demonstrated that increasing temperature increases transpiration rates in this species maintaining appreciable level of photosynthesis even at 35 °C.

On the contrary, at LT our ragweed plants were shorter and more laterally expanded, likely to avoid heat dispersion, as happen for perennial plants living at high latitudes or altitudes that usually grow in a cushion form to minimize the loss of heat and moisture [23].

In any case, although the shoot architecture, the growth rate and flowering were temperature-dependent (slower at LT), in our experimental conditions all the ragweed plants successfully completed their life cycle flowering and producing a similar final dry biomass. It suggests that common ragweed can diffuse in regions where the minimum and maximum temperature range between 14 and 26 °C and between 18 and 30 °C during the vegetative summer season. However, in warm climate the lack of seed stratification, due to too high winter temperature, may prevent germination and then the plant spreading [24]. Moreover, it must be taken into account that in many warm regions other different factors severely constrains *A. artemisiifolia* development such as the low rainfall during the summer months in Mediterranean area [25, 26]. Then, even if common ragweed can tolerate high temperature it is unlikely that it may spread in warm climate region with high winter temperature and/or low rainfall during vegetative season. On the other hand, a further prolonged pollen season may be expected in the already suitable climatic regions for common ragweed where the species is naturalized or invasive, raising the period of exposure to allergens. Furthermore, in those regions such as Central/Norther Europe and mountains, where now common ragweed cannot complete its life cycle because of the mean low temperature of the vegetative summer season [15, 27], the global warming may allow its growth and reproduction extending the area of allergen exposure. In this type of environments, although germination may be partially affected by low temperature (Table 1), the currently casual common ragweed populations may become naturalized and even invasive because temperature would be no more the principal climatic limiting factor. This agrees with all the distribution models developed for *A. artemisiifolia* [15–17] predicting that this species will expand globally and specifically northward and uphill in Europe increasing the allergens-exposure areas. In addition, we observed a higher production of male inflorescence at LT. It should increase pollen production in relatively cold climates contributing to enhance subject exposure to allergens. Nevertheless, our results about pollen allergenicity show that it is lower at LT, decreasing the expected impact on allergy rise determined on the basis of the sole plant distribution and pollen production. In fact, in addition to the temporal and spatial magnitude of the exposure, pollen allergenicity is widely recognized as a major determinant of the prevalence and/or severity of allergic diseases [8].

Previously, Ghiani et al. [11] demonstrated that the allergenic potency of ragweed pollen is mainly governed by climatic changes occurring during plant development and flowering. By our experiment, we confirmed that pollen allergenicity is an epigenetic controlled trait and we demonstrated that it is highly responsive to temperature. Both the synthesis of allergenic proteins and the Amb a 1 – IgE binding were governed by ambient temperature leading to a positive correlation between total pollen allergenicity and temperature.

Specifically, the major changes in allergen synthesis between LT and HT pollen were related to the enolase (Amb a 12), and UDP-glucose pyrophosphorylase-like proteins, that were highly expressed in LT pollen, and to a desiccation related protein PCC13–62-like that was more abundant in LT pollen as well.

The synthesis of all these allergenic proteins was previously demonstrated to be enhanced by low temperature and likely involved in temperature change tolerance [28–30]. Particularly, enolase was demonstrated to act as a positive regulator of cold-responsive gene transcription in *Arabidopsis thaliana*. It functions as a transcriptional repressor of STZ/ZAT10, which is a repressor of cold-inducible CBF (C-repeat binding factor) pathway [28, 31]. Among the cold-responsive genes, the other two allergenic proteins mainly synthetized in LT pollen, the UDP-glucose pyrophosphorylase and the desiccation related proteins PCC13–62, are listed. They were described as regulatory factors closely involved in the homoeostatic readjustment of plant responses to environmental signals [29, 32–34]. UDP-glucose pyrophosphorylase is a key enzyme producing UDP-glucose, which is involved in an array of metabolic pathways concerned with, among other functions, the synthesis of sucrose and cellulose. It was also suggested to play a critical role in plant growth and reproduction [32–34]. PCC13–62 proteins were instead first identified in the resurrection plant *Craterostigma plantagineum* and suggested to act in plant desiccation tolerance. However both the protein families appear to respond to a broad range of adverse environmental conditions and their specific role still needs to be clarified [30]. In any case, the increased synthesis of these three allergens in LT pollen should have increased its total allergenic potency, but this was not the case in our experiments because in LT pollen the major *A. artemisiifolia* allergen, Amb a 1, although normally synthetized, was only partially recognized by specific IgE; the result was a lower total allergenicity than the one measured for HT pollen normally showing the usual Amb a 1 - IgE binding.

Pollen flavonoids were identified as the responsible factor for the reduction of the Amb a 1 - IgE binding: the pollen concentration of flavonoids increased with the decrease of temperature and was positively correlated to the increase of allergenicity (Fig. 3 and Additional file 1: Figure S1). Moreover, the addition of the flavonoid rutin to pollen extracts led to decreased allergenicity in a concentration-dependent manner (Fig. 4).

Interestingly, literature suggests that quercetin-type flavonols are implicated in temperature change responses and their concentration increases at low temperature [35, 36]. Moreover it suggests that quercetin-type flavonols naturally bind some allergens such as the strawberry and birch pathogenesis-related 10 (PR-10) proteins Fra a 1 and Bet v 1, inducing protein conformational changes in critical loop regions [19, 37]. However the relevance of these interactions both for plant funtion and allergenic potential needs to be further studied. Indeed, concerning the influence on allergenic potency, Seutter von Loetzen et al. [38] investigated the role of quercetin-type ligands on Bet v 1 allergenicity showing that ligand binding to three different isoforms of Bet v 1, strikingly different in their immunological and allergenic properties, is diverse and highly dependent on the composition of their sugar moieties. However, although conformational changes of Bet v 1 structure were observed, the authors did not find a direct ligand effect on IgE recognition of Bet v 1, opening the possibility of indirect influences on sensitization in their experimental context. On the contrary, we observed a clear and significant influence of quercetin-type flavonoids on the Amb a 1 - IgE binding. We can speculate that the association of high amounts of flavonoid with Amb a 1 may have covered allergen epitopes and/or induced Amb a 1 structural conformational changes that altered the epitopes partially affecting IgE recognition. Anyway, further experimental proofs and studies are needed to determine the biological role of flavonoids – Amb a 1 complexes in plant under normal and ambient temperature changes. At this regards, noteworthy, Casañal et al. [37] reported that Fra a 1 proteins control flavonoid biosynthesis through binding to metabolic intermediates. As flavonoids participate in many aspects of plant biology including pollen germination and cell protection during biotic and abiotic stresses, we can speculate that the pectate lyase Amb a 1 binds intermediates of flavonoid biosynthesis to modulate the amount of these secondary metabolites within pollen to face ambient thermal changes. Furthermore, based on the hypothesis by Seutter von Loetzen et al. [38] suggesting a role for Bet v 1:querceitin-type-flavonols complexes in recognition processes during fertilization, Amb a 1 - querceitin-type flavonols complexes may also have specific roles in common ragweed reproduction. Experiments to clarify the role of the Amb a 1-rutin complexes are underway.

## Conclusions

Overall, our results show that *A. artemisiifolia* responds to temperature variation mainly by changing shoot architecture and by modulating growth rate, the number of male inflorescences and the synthesis of pollen allergenic proteins and flavonoids. Although the molecular mechanisms are still to be clarified and further experiments are needed, these morpho-functional modifications make the species tolerant to environmental temperature changes favoring its spreading and reproduction under temperature variations. As a consequence, such global change factor strongly influences the prevalence and severity of *A. artemisiifolia* allergy by modulating not only the temporal and spatial magnitude of subject exposure to pollen but also the allergenic potency of pollen itself. It support the idea that the ongoing climate change will increase the global ragweed impact on allergy, although specific experiments testing the genetic adaptation of ragweed to new ambient temperatures are needed to understand the long-term effect of climate change on allergy.

## Methods

### Plant growth conditions and traits measurement

*Ambrosia artemisiifolia* seeds from a rural site near Milan, Italy (45°35′59.0" N; 8°52’29.0" E) were cold stratified at 4 °C for 3 months under continuous darkness, sterilized for 10 min in 5% sodium hypochlorite, rinsed with three changes of sterile distilled water and then germinated and grown in controlled conditions. Three growth chambers with identical and constant photoperiod, light intensity (15:9 h light:dark 150 μmol m^− 2^ s^− 1^) and humidity (65%) but different temperature (LT: 18–14 °C light-dark, IT: 24–20 °C and HT: 30–26 °C), were used.

Germination tests were set up inside each growth chamber and performed in 1% (*w*/*v*) plant agar (Duchefa, The Netherlands). For each temperature, 150 seeds were tested in five distinct Petri dishes and the percentage of germination was calculated after 6 weeks. In parallel, inside each growth chamber, about 300 seeds were sown in 10% organic matter soil, pH 6–6.5 and left to germinate.

For each temperature fifty-one two-leafed (cotyledonary) stage seedlings were transferred to pot trays containing the same soil and the plantlets were grown for about 4 months until seed setting. During plant development data about vegetative and reproductive traits were collected: the maximum plant height and lateral expansion, the number of male inflorescences and female flowers were weekly measured whereas the plant aerial biomass was assessed at the end of growth by measuring shoot dry weight. The starting of flowering was also assessed on the basis of the emission of the first and second male inflorescence and female flower.

Mature pollen was collected from 20 LT, 25 IT and 19 HT plants by covering each male inflorescence with a transparent plastic collector according to Ghiani et al. [11]. Sampled pollen was stores in 2 ml tubes in boxes containing silica gel at room temperature until use.

### Protein slot blot

Slot blot technique was applied to assess the whole allergenicity of pollen collected from single plants and was carried out according to Ghiani et al. [14]. Briefly, soluble pollen protein extracts were prepared by suspending 0.1 g pollen in 1 ml of bidistilled sterile water containing protease inhibitor (1 mmol L^− 1^ phenylmethylsulfonyl fluoride). Sample were incubated on a rotating drum for 3 h at room temperature. The soluble fraction was isolated by means of two centrifugations at 13000 RCF for 10 min at 4 °C and then stored at − 20 °C until use. Protein concentration was assayed according to Bradford [39] using bovine serum albumin (BSA) as standard. At least three independent pollen extracts were prepared for each plant. Equal volumes of protein extracts were bound to nitrocellulose membrane and first stained with Ponceau S staining solution [0.1% (*w*/*v*) Ponceau S in 5% (*v*/v) acetic acid] to assess the amount of proteins loaded in each well. After the removing of Ponceau S staining, membranes were used to evaluate the immunoreactivity of the different pollen extracts to a pool of sera from adult subjects allergic to common ragweed. The pool of sera was previously prepared by mixing 12 sera selected for their ability to specifically detect ragweed allergens [11]. All together, the 12 sera can bind nearly all the common ragweed allergens and allow to detect the differences among pollen samples [11].

The study was based on data stemming from routine clinical activity and on stored sera previously used to perform routine clinical investigations; the study has been approved by the Institutional Review Board. For this purpose membranes were blocked with 5% (w/v) non-fat dry milk powder in TBS-T [20 mM Tris, 150 mM NaCl and 0.05% (v/v) Tween 20, pH 7.5] for 1 h and then incubated for 16 h at 4 °C with a 1:10 dilution of the mixed sera from ragweed-allergic patients. Bound IgE were detected using an HRP-conjugated goat anti-human IgE antibody (1:15000 dilution; Sigma). Protein extract from commercial pollen (Allergon) was used as standard to control staining variation when comparing measurements referring to different experiments. Negative controls were performed by omitting the sera mix and by using a pool of sera from non-atopic subjects. Immunoreactive spots were visualized on an X-ray film (Kodak) using Amersham ECL prime western blotting detection reagent (GE Healthcare). Image analysis was applied to quantify immunochemical signals: the integrated optical density (IOD) of immunoreactive spots with respect to the IOD of standard (sample IOD/standard IOD) was measured. The mean results of five independent experiments were calculated and statistically analyzed by applying Kruskal-Wallis non-parametric procedure.

Slot blot technique was also applied to assess the effect of rutin (quercetin-3-O-rutinoside) on pollen allergenicity. For this purpose 2 μl of commercial pollen extract containing 3 μg of proteins was mixed with increasing amounts of rutin (ranging from 0 to 3,2 μg), bound to nitrocellulose membrane and treated in order to assess the reaction with the pooled sera as above reported.

### One and two dimensional immunoblotting

1D and 2D immunoblot analyses were carried out to study the effect of temperature on allergen profile.

For 1D immunoblot analysis, pollen extracts were directly dissolved in SDS sample buffer [2% (*w*/*v*) SDS, 10% (*v*/v) glycerol, 1 mM DTT, 62.5 mM Tris-HCl, pH 6.8], wheras for 2D immunoblotting, extracts were first purified with a clean-up kit (Bio-Rad Laboratories®) and finally dissolved in IEF rehydration buffer (7 M urea, 2 M thiourea, 2% (w/v) CHAPS, 20 mM Tris–HCl, pH 8.8, 20 mM DTT, 0.5% ampholyte mixture carrier, pH 3–10, 0.005% bromo-phenol blue). One-D immunoblotting was carried out following the protocol reported by Aina et al. [40]. Briefly, equal amounts of proteins (30 μg/lane) were separated by 14% SDS-polyacrylamide gels according to Laemmli [41]. Gels were either stained with colloidal Coomassie Blue G-250 (0.1% Coomassie Blue G250, 170 g/l ammonium sulphate, 34% methanol, 3% phosphoric acid) or transferred to nitrocellulose membrane. Nitrocellulose filter saturation and sera-mix reaction was performed as reported above for slot blotting. For each temperature, at least 1 sample from each plant and 5 independent samples prepared by mixing pollen extracts from all the plants were analyzed.

Two-D immunoblotting was performed according to Asero et al. [42]. Isoelectrofocusing (IEF) was carried out on 11 cm long immobilized pH gradient (IPG) strips (Bio-Rad®), providing a linear pH 4–7 gradient. Strips were rehydrated in 200 μl of IEF rehydration buffer containing 70 μg of protein sample. Passive rehydration and IEF were performed at 20 °C using a Protean IEF-Cell (Bio-Rad Laboratories®). After the first dimension separation, the IPG strips were equilibrated for 15 min against 6 M urea, 30% glycerol, 2% SDS, 0.375 M Tris–HCl pH 8.8, 2% DTT, in order to resolubilize proteins and reduce disulfur bonds. The –SH groups were then blocked by substituting the DTT with 2.5% iodoacetamide in the equilibration buffer for 15 min. After equilibration, strips were placed on the top of vertical polyacrylamide gels (14%). An agarose solution (0.5% low melting agarose in running buffer) was loaded to the top of the gel to lock strip and electrophoresis was performed at 4 °C in a Laemmli running buffer (25 mM Tris–HCl pH 8.3, 192 mM glycine, 0.1% SDS). Gels were run in parallel and used for protein revealing or immunoblotting experiments. Protein staining and immunoblotting were performed as above reported for 1D experiments. For each temperature, at least 3 independent samples prepared by mixing pollen extracts from all the plants were analyzed.

In order to identify IgE bound proteins, immunoreactive bands were carefully excised from Coomassie-stained 1D and 2D gels, submitted to in-gel trypsin digestion and the obtained tryptic fragments analyzed by LC-MS/MS according to Asero et al. [42].

### Determination of flavonoid content

The aluminium chloride colorimetric method was used to determine the concentration of flavonoids in aqueous pollen extracts according to the protocol of Pękal & Pyrzynska [43]. Pollen extract (0.2 ml) was mixed with 0.06 ml of NaNO_2_ (5% *w*/*v*) and after 5 min, 0.06 ml of AlCl_3_ (10% w/v) were added. After further 6 min, the sample was neutralized with 0.4 ml of 1 M NaOH solution and incubated for 10 min at room temperature, after which the absorbance at 510 nm was measured. Quercetin was used as standard, with a linear calibration curve ranging 10–250 μg/mL and results were expressed as milligrams of quercetin equivalents per gram of pollen. All measurements were conducted in triplicates.

### Statistical analysis

Statistical analyses were performed by GraphPad Prism software for Windows (version 4.0 GraphPad Software Inc., San Diego CA): ANOVA and Tukey test were applied to the data when normality and homogeneity of variance were satisfied (plant height, lateral expansion, dry biomass, flavonoid content). Data not conforming to the assumption were analysed by Kruskal-Wallis non-parametric procedure (allergenicity, number of flowers, flowering). Chi-square test was applied for seed germination. R software, version 3.3.2 [44] was also used to perform regression analyses.

## Additional file

Additional file 1: **Figure S1**. Flavonoid content and total allergenicity of pollen from *A. artemisiifolia* single plants grown at different temperatures. (DOCX 2774 kb)

## Abbreviations

1D: One-Dimensional
2D: Two-dimensional
BSA: bovine serum albumin
CBF: C-repeat binding factor
CHAPS: 3-[(3-Cholamidopropyl) dimethylammonio]-1-propanesulfonate hydrate
DTT: Dithiothreitol
ECL: Electrochemiluminescence
HRP: Horseradish Peroxidase
HT: High Temperature
IEF: Isoelectric focusing
IgE: Immunoglobulin E
IOD: Integrated Optical Density
IPG: immobilized pH gradient
IT: Intermediate Temperature
IUIS: International Union of Immunological Societies
LC-MS/MS: Liquid chromatography-tandem mass spectrometry
LT: Low Temperature
PR: pathogenesis-related
RCF: Relative Centrifugal Force
SDMs: Species Distribution Models
SDS-PAGE: Sodium Dodecyl Sulphate - PolyAcrylamide Gel Electrophoresis
UDP-glucose pyrophosphorylase: Uridine Diphosphate Glucose Pyrophosphorylase
