# Supplementary material for: Ambrosia artemisiifolia L. temperature-responsive traits influencing the prevalence and severity of pollinosis: a study in controlled conditions
Source: BMC Plant Biol. 2019 Apr 25;19:155. doi: 10.1186/s12870-019-1762-6 (PMC6482493; doi:10.1186/s12870-019-1762-6)

**Impact of temperature on Ambrosia artemisiifolia L. pollinosis: a study of plant temperature-responsive traits in controlled conditions**

Rodolfo Gentili, Riccardo Asero, Sara Caronni, Maria Guarino, Chiara Montagnani, Gianni Mistrello, Sandra Citterio

**Supplementary material: additional file 1**

**Fig. S1** Flavonoid content and total allergenicity of pollen from *A. artemisiifolia* single plants grown at different temperatures. (**a**) Representative picture showing the color of pollen extracts and their related allergenic potential determined by Slot blot. (**b**) Total flavonoids determined by aluminium chloride colorimetric method and total allergenicity determined by slot blot analysis in pollen from single plants. LT: Low Temperature (18-14°C light-dark), IT: Intermediate Temperature (24-20°C light-dark), HT: High Temperature (30-26°C light-dark). Numbers: plant ID.

**a**


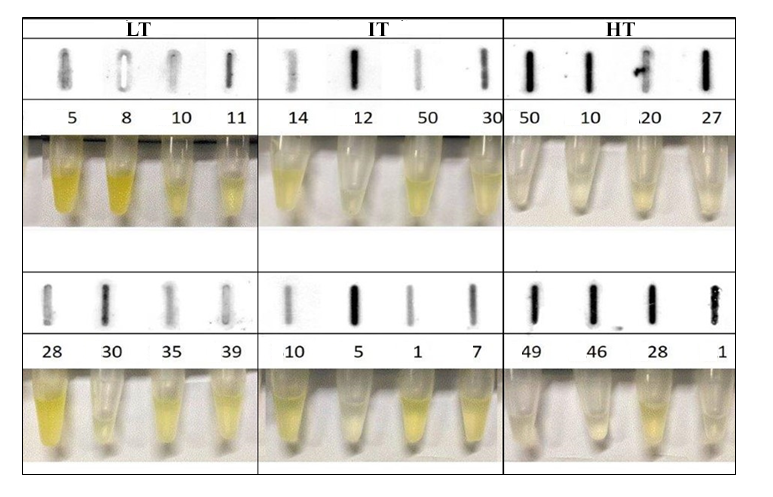


**b**


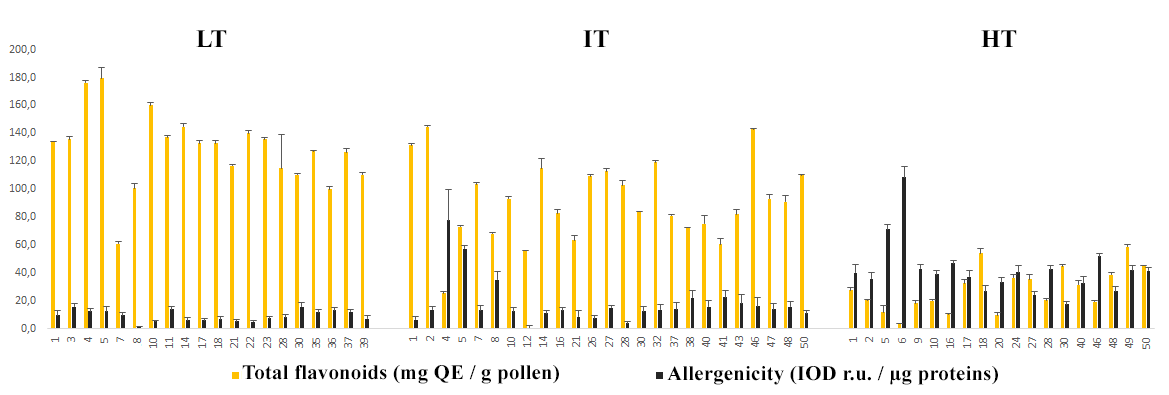

Supplement: Supplementary file 1 — Figure S1. Flavonoid content and total allergenicity of pollen from A. artemisiifolia single plants grown at different temperatures. (DOCX 2774 kb) [file 12870_2019_1762_MOESM1_ESM.docx]
